# Mothers discourses on baby monitors: A discursive analysis of Mumsnet posts on surveillance and risk management

**DOI:** 10.1177/13591053251353198

**Published:** 2025-08-20

**Authors:** Chloé Michoud, Abigail Locke, María del Río Carral

**Affiliations:** 1 Keele University, UK; 2 University of Lausanne, Switzerland

**Keywords:** critical health psychology, baby monitor, mothering practices, qualitative methods, sleep monitoring device

## Abstract

Despite recommendations against using commercial sleep monitoring devices to prevent sudden infant death syndrome (SIDS), their use is increasing among parents. This study explores mothers’ discursive constructions about the usage of baby monitors during their infants’ sleep. By analysing 43 online posts from the website Mumsnet through Critical Discursive Psychology, two key discourses were identified: ‘surveillance as a good mothering practice’ and ‘risk management via technology’. The first repertoire illustrated how mothers incorporate digital surveillance into their daily routines, using baby monitors as essential tools to enact ‘good mothering’. The second repertoire shows how technology is portrayed as a beneficial tool for mothers and parents, helping in childcare and enhancing safety. In conclusion, baby monitors are seen as essential for managing mothering risks, but their pervasive use can create challenges, raising concerns about whether mothers should adapt to these tools or if the tools should better serve parents’ needs.

## Digital devices and baby monitors

The expansion of contemporary surveillance and risk cultures has facilitated the widespread development of digital health-related monitoring devices (e.g. apps, wearables, bracelets, and watches) designed to track bodily states and health-related behaviours by capturing, quantifying and evaluating physical and physiological indicators (del Río Carral et al., 2017). Initially developed to address contemporary health challenges, these devices are aimed at promoting individual behaviour change, also offering unprecedented opportunities for continuous self- or other-monitoring (Lupton, 2016; Swan, 2012). From eating and exercising to walking, breathing and sleeping, most aspects of daily life can now be tracked through these technologies (Johnson et al., 2016; Lupton, 2012).

This rapid rise of health-related monitoring devices can be understood by considering contemporary forms of governance, particularly, neoliberalism (Foucault, 1988). With an emphasis upon the individual as voluntary, rational, and capable of self-surveillance, the neoliberal discourse encourages individuals to engage in continuous psychological and physical labour, to work towards self-improvement (Riley et al., 2018). In addition, health and healthcare services are treated as marketable goods or commodities, subject to the principles of supply and demand, rather than as public goods or rights (Lupton, 2012; Petersen and Lupton, 1996; Turrini, 2015). In this context, digital devices have become massively popular commodities that promote monitoring practices among individuals regarding health practices, often with the promise of becoming healthier and happier (Ahmed, 2010; Lupton, 2012).

Digital tools aimed at tracking infant sleep are advertised to new and expectant parents as providing possibilities for alleviating the challenges and concerns of caring for an infant and enhancing parental welfare. Such claims are supported by research conducted in digital technologies (e.g. Maurya et al., 2021). Baby monitoring devices are common in the form of onesies, socks, buttons, leg bands and diaper clips, measuring biological sign (e.g. pulse rate, respiration). According to Ball and Keegan (2022), they play four main roles: (a) to help parents better understand their infant’s sleep, (b) to support parents in providing night-time care, (c) to assist parents in ‘managing’ their baby’s sleep and (d) to facilitate remote monitoring of infant sleep’ (p. 1). Although all the products have a warning that they do not prevent sudden infant death syndrome (SIDS; Ball and Keegan, 2022), they are marketed as such.

Sleep-related infant deaths continue to represent a significant public health concern, known as SIDS. The predominant etiologic hypothesis posits that SIDS arises from the interplay among three distinct variables (Filiano and Kinney, 1994): underlying intrinsic vulnerability, a critical developmental period, and extrinsic factors in the infant’s environment (e.g. prone sleep position, bedding surfaces). SIDS is the leading cause of death among post neonatal (age 28 days to 11 months) infants (Salm Ward and Balfour, 2016), despite its major decrease after the ‘Back to Sleep’ campaign in 1991 (Leach et al., 1999), which promoted the supine position for infant sleep. Thanks to this initiative, the SIDS post-neonatal infant mortality rate decreased by 71.3%, whereas the overall SIDS infant mortality rate decreased by 70.9% between 1983 and 2012 (Goldstein et al., 2016). While some studies have shown that a breastfeeding duration of at least two months is associated with half the risk of SIDS (Thompson et al., 2017; breastfeeding does not need to be exclusive to confer this protection), the most important risk factors for SIDS relate to the sleep environment, particularly sleep position, which is a strong risk factor (Adams et al., 2015). Today, reductions in SIDS are commonly attributed to modifications in infant sleep environments (Goldstein et al., 2016). Therefore, parents receive multiple and varied efforts to promote adherence to safe sleep recommendations aimed at reducing the risk of sleep-related deaths, such as a clean cot, a firm, flat, waterproof mattress; keep the baby smoke-free; avoiding getting the baby too hot; sleeping in the same room as the baby for every sleep, day and night for at least six months (NHS, 2024; NIH, 2023; The Lullaby Trust, 2024). The American Association of Paediatricians recommends avoiding the use of sleep monitoring tools, as well as commercial devices marketed to reduce the risk of SIDS (Moon et al., 2022). However, (millennial) parents are embracing wearable smart devices as a means to prevent unexpected infant death or abnormal events (Hasan and Negulescu, 2020). Sales of these products remain strong and the market continues to grow, despite the absence of publicly accessible evidence confirming their safety, accuracy, effectiveness or their significance in the care of healthy infants (Bonafide et al., 2017).

## Infant sleep, risk and surveillance

While some studies emphasise the biological aspects of infant sleep development (Joseph et al., 2015), other scholars argue that concerns over infant sleep are shaped by cultural expectations and historical shifts in mothering norms (Giannotti and Cortesi, 2009; Keller and Goldberg, 2004). The present study builds on the perspective that infants’ sleep is constructed by specific discourses, social practices and advice (e.g. parental leave policies, paediatric guidelines, educational books and monitoring technologies).

Advice to parents and mothers about baby sleeping practices can be positioned within the field of Mothering Culture Studies (e.g. Lee et al., 2014) that examines how wider society shapes mothering norms and expectations. Such norms become part of intensive mothering ideologies (e.g. Hays, 1998) that portray ‘good mothering’ to be around adopting child-centred mothering practices, that is, mothering around the needs and interests of the child instead of those of the parent, making the child’s ‘voice’ the priority.

Tied to these ideologies, we see how ‘risk culture’ has become embedded in the broader discourses on mothering, for instance, during pregnancy, women experience heightened levels of surveillance due to carrying a child (e.g. Gross and Pattison, 2007; Lupton, 1993) with expectations that they will adopt health behaviours to protect the foetus. Other mothering decisions such as infant feeding (Knaak, 2010; Locke, 2015) or, as in the case of sleep practices, whether to co-sleep or room-share with the infant for the first few months (e.g. Keller and Goldberg, 2004), are framed within neoliberal notions of informed choice and risk. These framings are particularly evident in the context of infant sleep, where the societal context of surveillance plays a key role in managing the ‘risks’ associated with different sleeping practices (Jewitt et al., 2021). One example of this heightened surveillance is the widespread use of baby monitors, which contribute to increased maternal vigilance.

While some parents reject monitoring technologies, scholars have underlined a broader ‘dataveillance’ trend (Lupton and Williamson, 2017) in which the infant body is an object of surveillance, measurement and monitoring. Despite the widespread adoption of baby monitors, some scholars argue that these devices reinforce anxieties rather than alleviate them, potentially leading to increased parental stress (Jewitt et al., 2021). Others suggest that reliance on such tools may create a false sense of security, reducing adherence to established safe sleep practices (Ball and Keegan, 2022).

Building on these discussions of surveillance and risk, this study explores how mothers navigate these tensions through their use of baby monitors, particularly within online forums. While part of the literature refers to ‘parents’, we choose to specifically focus on mothers, as they are typically responsible for most of the caregiving and domestic tasks within families. In the United Kingdom, women are more likely to become carers and to provide more hours of unpaid care than men (Petrillo et al., 2022). It is therefore essential to examine ways in which mothers make sense of monitoring devices regarding health-related practices (Rio Carral et al., 2016), in order to understand the ways in which it co-constructs expectations concerning contemporary motherhood.

## Method

Adopting a sociocial constructionist approach, our research investigates how mothers discuss the role of digital devices in monitoring infants’ sleep. This includes examining how mothers discursively construct their use of these technologies within online forum and analyse discourses surrounding infants sleep monitoring.

### Data

To explore online discursive constructions around this topic, the popular website in the United Kingdom, Mumsnet, was searched using the term ‘Baby Monitor’ to identify posts about the use of baby monitors. This search, conducted between 13 March and 12 July 2024, identified 316 posts related to baby monitors in various ‘Talk’ sections of Mumsnet, published between 14 August 2009 and 26 June 2024. Given the focus of the research on contemporary parenting discourses, the analysis only considered posts published after January 2015, of which there were 198. These posts were classified inductively according to their content, and three distinct types of post were identified – (1) Technical  +  Recommendation (147) posts focusing on particular technical aspect of the device as well as a recommendation of which type of monitor to buy; (2) Advertisement (8); and (3) Use of Device (43)). Focusing on how mothers navigate baby monitor practices, the ‘Use of Device’ posts were selected for further analysis. By highlighting practical challenges and questions, these posts offer direct insights into how mothers interact with baby monitors in their daily lives. We analysed the ‘original post’ exclusively, not the replies stemming from this primary message. Examining only the posts allowed us to better identify the dominant discourses underlying mothers’ interactions with baby monitors. Indeed, this focus helps identify the primary concerns, subject positions and discursive accomplishments that the mothers construct around these devices without the added layer of interpretation that replies might bring. This resulted in 43 posts that are presented in the analysis.

### Ethics

All data included in this research are freely posted into the public domain where there is an expectation that they will be shared and commented upon. However, the British Psychological Society argues ‘Participants may not be fully aware of the degree to which their discussion group posts are already available to public scrutiny […]’, meaning that research should be extremely careful with ‘detailed personal narratives’ (British Psychological Society, 2017: 11). Following that recommendation, all identifying information including pseudonyms, region/country and profile photographs have been removed to protect users’ identity. Ethical approval was granted from the Research Ethics Ethics Commission of the University of Lausanne (CH) project n° C-SSP-012024_00006. Further, a notification of data usage was sent to Mumsnet to inform them about the research project. Mumsnet granted permission for the academic use of relevant posts, provided they are acknowledged and personal identifiers are removed.

### Approach and analysis

This study used Critical Discursive Psychology (Locke and Budds, 2020; Wetherell, 1998) to analyse the data set. This methodology was selected due to its dual focus on both macro and micro levels of analysis which made it ideal for addressing online textual content setting it within wider mothering contexts. The initial data set was coded by the first author, following the steps set out by Locke and Budds (2020). This meant that initially posts were coded simply for their content, which helped to identify posts with questions or opinions around the use of baby monitors. This allowed to further identify posts that were useful for the analysis. Following that stage, the first and second authors identified constructions of the topic of investigation, for example the idea of constant surveillance using the baby monitor. The next step focused on identifying interpretive repertoires, that is, recognisable building blocks of discourse within the data. Stage four aims at identifying subject positions (e.g. a subject position of ‘good parent’ can be identified in most of the posts analysed when the poster adopts a discourse of risk prevention). In the last stage, the posts were coded to examine what strategies and features were used to accomplish particular interactional effects.

Four posts are presented in the analysis. Other than anonymising authors, the posts are shown as they originally appeared, including any typographic errors. Furthermore, we have assumed that the posts on Mumsnet were written by mothers. While we recognise that Mumsnet is accessible to all and may include contributions from fathers, men, non-binary individuals or transgender people, we have presumed it is predominantly used by women. The name of the site itself signals its focus on mothers and their caregiving roles. We have chosen to reflect this context in our data analysis, acknowledging the experiences and labour of women.

## Findings

Use of the baby monitors were often diverse, with mothers constructing various personal, relational and circumstantial uses of such devices, shaping their digital practices in many ways. Baby monitor practices were narrated as crucial, sometimes complicated and demanding. Mothers often constructed baby monitors in terms of surveillance and ‘preventing the worse’. Primarily, they drew upon two different interpretative repertoires: (1) surveillance as a good mothering practice, and (2) risk management via technology. Both repertoires are evident in the posts under analysis. Additionally, the posts analysed are presented in an evolving frame, reflecting how the use of baby monitors shifts over time – from early discussions about providing peace of mind to later reflections on when to stop using the devices, capturing the changing framing of digital surveillance across different stages of mothering.

### Post 1 – Is a baby monitor helping for peace of mind?

The first extract begins with a post from a pregnant woman who is looking for opinions and experiences with audio or connected mattress baby monitors.

1. I'm due my first baby at the end of January, and the only thing we have left to

2. purchase is the monitor. I'm torn between an audio and one that has the motion mat.

3. Before anyone states…I know it doesn't prevent SIDS…but coming from a family who

4. has experienced cot death, I think it just adds a little reassurance, and think I would

5. sleep a little better knowing that there is that extra thing in place. I guess I would

6. rather a false alarm than waking up and finding the worse!

7. I know I have read that they give what people think are false alarms, but some say the

8. noise is enough to startle baby, so can't be sure it's definitely a false alarm.

9. I know I don't want a video one as I would sit watching it constant and wouldn't relax.

10. I would like to see what people's opinions/experience is with the audio or motion

11. monitors…would you purchase same again?

In this first extract, we can see both repertoires at play. The ‘risk management by technology’ is evident as the poster states that despite knowing that monitors do not prevent SIDS, there is still an element of managing this risk through having one but instead sets out their reasons of planning ahead, knowledge and taking preventive measures to avoid ‘finding the worse’ (line 6), that is presumably, a dead baby. The poster outlines her close experience of being from a family who has experienced cot death. In discursive terms, note here the use of ‘worse’ as an extreme case formulation (Pomerantz, 1986) to demonstrate the extremity and obvious difficulty of that event. The expectant mother shows her alignment with these discourses by meticulously considering her options for baby monitors, a decision she frames as crucial to ensuring her future child’s safety. By repeating ‘I know’ three times (lines 3, 7, 9) she positions herself as an informed and knowledgeable parent, while also acknowledging the limitations of the device, therefore underscoring the value she places on any additional reassurance it might provide. This awareness and planning, despite the known limitations of the motion mat monitor, are portrayed as essential aspects of responsible mothering and demonstrate the second interpretative repertoire of ‘surveillance as a good mothering practice’. Her preference for a device that might give ‘false alarms’ reflects a broader mothering ideology where taking preventive action – even in the face of potential inconvenience – serves as a safeguard against potential unforeseen tragedies. This perspective reinforces the notion that ‘good’ mothering involves being well-informed, proactive, and willing to endure minor disturbances to avoid the catastrophic ‘worse’ outcomes.

Simultaneously, the mother manages her accountability by employing softeners (Edwards, 2000) and hedging language to justify her choices, thereby preventing potential criticism. She uses phrases like ‘just adds a little reassurance’ (line 4) and ‘sleep a little better’ (line 5) to temper her decision to purchase a motion mat, which is known for producing false alarms and has received negative reviews. These softeners serve to downplay the potential faults of the device, framing it as a small, manageable addition rather than a significant source of stress or anxiety. In doing so, she subtly mitigates her accountability for any potential disruptions caused by the device, positioning herself as a cautious and thoughtful mother who is simply taking an ‘extra thing’ (line 5) for her child’s safety. This careful balancing act reflects the broader cultural expectations surrounding ‘good mothering’, where mothers are expected to perform due diligence and make informed decisions for their children’s well-being. The mother’s inquiry about the experiences and opinions of others regarding different types of monitors also suggests a communal aspect to these discourses, where mothering choices are validated and refined through shared knowledge, further emphasising the importance of informed decision-making in the pursuit of being a ‘good’ mother.

### Post 2 – Is it safe to let baby alone with a baby monitor?

This second post starts with an explanation of the evening/sleeping routine adopted and asking for advice and examples of others’ routine regarding baby sleep. The underlying question could be formulated as ‘Is it safe to let a baby sleep alone before 6 months under the surveillance of the baby monitor?’ As with the first post, both interpretative repertoires are evident within this second post.

1. Hi! Just curious of what others do on this.

2. My son in 4 months old. We start to wind him down around 7pm, pjs, bottle, story

3. time etc then by 9pm he's ready for bed, we put him up the stairs in our bedroom in

4. a next to me crib with a baby monitor on, we sit down stairs to watch TV for a bit

5. with a cuppa then head up a couple of hours later, the monitor is on and we can see

6. him clearly in it, I've read a few things that you shouldn't be putting a baby to bed

7. alone until they are 6 months old, any advice would be much appreciated

8. Thanks x

In this second extract, the mother begins by using a softener of ‘just curious’ (line 1) (Edwards, 2000) to ask other users about their mothering practice which works as a discursive strategy to align themselves with a generalised norm. By positioning their question as a casual curiosity rather than a serious concern, she reduces the perceived weight of their actions and subtly distances themselves from any potential criticism. This approach is further reinforced by ‘others’ (line 1) which serves to align the speaker with the broader ‘parents’ group, suggesting that her practices are part of a common, socially accepted routine.

The management of potential accountability is a key theme in this text, as the mother carefully navigates the potential criticism of her actions (not following official advice) by highlighting the shared nature of mothering responsibilities while also emphasising the personal commitment to caring for her child. The use of ‘we’ in phrases like ‘we start to wind him down’ (line 2) and ‘we put him up the stairs’ (line 3) indicates that both parents are involved in the child’s routine, suggesting a partnership in the mothering process (Locke, 2023). However, the use of ‘my son’ (line 2) when discussing the child’s bedtime routine subtly places the mother in a more central, caring role, thus reinforcing her responsibility and accountability for the child’s well-being. This is further evidenced by her mention of reading guidelines on sleep and the fact that she is writing the post to ask for advice, which positions her as a conscientious, informed mother and, therefore a ‘good mother’, who is actively seeking to do what is best for her child.

The discourse of ‘good mothering’ within this narrative is closely tied to the establishment and adherence to a routine, which is emphasised with a three-part list (Jefferson, 1990): ‘pjs, bottle, story time’ (lines 2–3). The three-part list serves to showcase specific, repeatable actions that are presented as a routine. By enumerating these actions – ‘pjs, bottle, story time’ – the list reinforces the idea that such routines are fundamental to establishing stability and care, key attributes associated with responsible mothering in this discourse. This structured approach reflects the expectation that ‘good mothering’ involves the creation and maintenance of consistent routines for children. The mention of the next-to-me crib and the use of a baby monitor to ensure the baby’s safety while the parents are downstairs further reinforces this discourse, highlighting the importance of vigilance and careful planning in mothering practices.

Moreover, ‘good mothering’ is also framed as a matter of limiting risk, particularly through the portrayal of the parents’ evening activities. By describing how they ‘sit downstairs to watch TV for a bit with a cuppa’ (lines 4–5) the mother contrasts this behaviour with potentially less responsible alternatives. This choice of words suggests that ‘good mothering’ involves moderation and a conscious effort to maintain a balance between relaxation and responsibility. The casual tone and the emphasis on watching ‘a bit’ of TV indicate that the parents are mindful of not overindulging, thereby maintaining their vigilance and readiness to respond to the baby if needed. The inclusion of the baby monitor in this scenario, with the mother noting that ‘we can see him clearly in it’ (lines 5–6) further underscores the discourse of ‘good mothering’ as encompassing surveillance and active monitoring, even when engaging in leisure activities.

The mother continues that she has ‘read a few things’ (line 6) about not putting babies to bed alone before 6 months, therefore framing the request for others’ perspectives. By presenting her actions as informed by but not strictly bound to guidelines, she manages the delicate balance between following expert recommendations and making personalised decisions based on their specific circumstances. As with the first post, we can see both of the interpretative repertoires to an extent within this post, for instance, the mother is advocating the use of a baby monitor (‘surveillance as a good mothering practice’ repertoire) as well as noted its use in managing safe baby sleep (‘risk management via technology’ repertoire).

Whilst these first two posts concerned advice over what type of monitor to use and whether to leave the baby to sleep upstairs in the evening with a monitor whilst the parents stayed downstairs until bedtime, there were also posts that asked about when it was appropriate to stop using baby monitors and what happens when monitors were not working as planned and giving false alarms. These next two posts focus in on this specific issue. Rather than being in opposition to the initial two posts, the two interpretative repertoires are evident.

### Post 3 – Asking permission to stop using the baby monitor

In this third post, the user is clearly expressing doubts about the use of a particular baby monitor which has a movement sensor. The poster states that it is going off several times during the night and is disturbing their and their partner’s sleep and seeking advice on what to do next.

1. Anyone else had this issue? We have the Angelcare sensor pad monitor for our

2. 3 month old and it’s going off several times during the night, I’ve turned the

3. sensitivity up to maximum (4) and it’s still doing it. She’s in a small cot in our

4. room so I’m generally getting to her before the warning beep turns to full alarm

5. and every time she’s breathing normally, quietly, obviously in a deep sleep but

6. she’s not snorting herself awake startled by it or anything. I kind of wish I’d never

7. bought it but now I darent not use it as what in case she really wasn’t breathing?!

8. we had the previous model for our first baby and never had an issue, couple of

9. alarms but when she was bigger and had crawled to the edge of the big cot. Not sure

10. what to do now!

In this post, the mother navigates accountability through the interplay of gendered tasks and personal responsibility, reflected in their alternating use of ‘I’ and ‘we’. The collective ‘we’ appears in references to shared decisions, such as ‘We have the Angelcare sensor pad monitor’ (line 1) and ‘We had the previous model for our first baby’ (line 8) distributing accountability between both parents. The mother mentions that the baby sleeps in a small cot in the parents’ room (lines 3–4), allowing her to intervene before the alarm fully sounds, which reassures both herself and potential critics of her attentiveness, as well as demonstrating that she is following official safety advice. Additionally, she references that with the previous model used on her previous child that they ‘never had an issue’ (line 8). This past success supports her decision to rely on the technology, framing the current problems as an anomaly rather than a failure of judgement.

However, she also expresses ambivalence, noting, ‘I kind of wish I’d never bought it but now I darent not use it’ (line 6). This statement shows the emotional burden of persistent false alarms and the fear of discontinuing the device. The phrase ‘darent not use it as what in case she really wasn’t breathing?!’ (line 7) encapsulates the technology-constructed anxiety, transforming what should be a source of reassurance into a cause of stress. This emotionally charged language underscores the ‘technology imperative’ where certain devices become seen as essential to ‘good mothering’, despite their potential to increase anxiety. As such here, both interpretative repertoires are alive in this extract in that the poster understands how surveillance of baby sleep is evidence of ‘good mothering’ and notes that they did this with their previous child also, whilst also noting the use of baby monitors in managing risk. There is no explicit question from the poster as to whether to continue to use a monitor, but there is a concern as to whether they continue to use this monitor. The request for input from others illustrates the complexities of accountability in mothering, where the use of technology is both a reassurance and a source of stress. Through this narrative, the mother manages her accountability and asks for guidance on what to do given the false alarms and disrupted sleep.

The final post (post 4) picks up on the issue of disrupted sleep and asks explicitly when is an appropriate time to stop using a baby monitor.

### Post 4 – The baby mon is disturbing parents’ sleep

This post starts with the mother describing their current sleep arrangement and asking if other parents relate to them. In the next paragraph, they describe the baby monitor as disturbing their sleep as it turns on when the baby is babbling away to sleep. The mother therefore asks other users if they are also experiencing this and at what age it is appropriate to stop the monitor.

1. My DS is 16 months old and has been sleeping in his own bedroom since he was about

2. 8/9months. Since then, my husband and I keep the baby monitor on in our bedroom all

3. night to make sure we can see/hear our DS. But I’m wondering if this is what

4. everyone does?

5. DS wakes most nights for a short period, he doesn’t cry or fuss, just babbles away to

6. himself before settling himself back to sleep, sometimes we have to pop in to put his

7. dummy back in reach. But I feel like it’s disturbing mine and my husbands sleep so

8. wondered at what age you stopped doing this (if you did it at all?!)

9. Thanks xx

This fourth extract, while quite similar to post 2, shows some notable differences. Here, the mother demonstrates the same ‘good mothering’ practices by emphasising the importance of surveillance and adherence to guidelines, noting in their mothering practice that these guidelines were followed, as the baby has been in his own room since 8/9 months (lines 1–2), which is more than the recommended 6 months. However, she also subtly seeks advice on modifying her current practices. This reference to a specific timeframe aligns with widely accepted mothering practices and guidelines, positioning the mother as knowledgeable and conscientious. By indicating that her child has been in his own room since a recommended age, she subtly justifies her decision and aligns herself with normative mothering standards. This adherence to guidelines further reinforces her identity as a ‘good parent’ who makes informed decisions based on established norms.

The post continues with a focus on surveillance, as the mother notes that ‘my husband and I keep the baby monitor on in our bedroom all night to make sure we can see/hear our DS’ (lines 2–3). This statement highlights again the expectation that ‘good mothering’ involves constant vigilance, particularly during the night when the child is in theirs’s or another room. The use of the baby monitor is portrayed as a crucial tool for ensuring the child’s safety and well-being, reinforcing the idea that responsible parents must maintain a watchful eye even as they sleep, similar to post two where parents are able to watch their child while they are watching TV.

However, as the text proceeds, she starts discussing the potential need to stop using the baby monitor, noting the infant’s age as an inferred justification (16 months). As she progresses with this inquiry, she constructs her account with more softeners and generalised language. Softeners, such as ‘if you did it at all’ (line 8) and ‘I feel like it is disturbing’ (line 7) help to manage accountability and mitigates potential criticism for considering a change in routine, presenting the issue as a matter of personal discomfort rather than a direct challenge to established mothering norms. The text also employs the use of generalised language, particularly with the phrase ‘I’m wondering if this is what everyone does?’ (lines 3–4). This use of ‘everyone’ makes the parent’s concerns relatable to a broader audience, managing accountability by framing the issue as a common dilemma rather than a unique personal problem.

Similarly to posts 2 and 3, accountability is carefully managed throughout the text, particularly with pronouns that shift between ‘we’ and ‘I’. The mother presents the monitoring of her child as a shared responsibility, using ‘my husband and I’ (line 2) to describe their joint efforts in keeping the baby monitor on at night. This collective approach distributes accountability between both parents, suggesting that the decision to use the monitor is a mutual one. However, as with previous posts, the text progresses, the pronouns shift to ‘I’ particularly when the mother expresses uncertainty and seeks advice (e.g. ‘I feel like it’s disturbing mine and my husband’s sleep’, line 7). This shift indicates that, while monitoring the child is a shared task, the emotional burden and the decision-making process may rest more heavily on the mother (Locke, 2023). This dynamic is further emphasised using ‘my son’ rather than ‘our son’, placing the mother in a more central, caring role, thereby enhancing her accountability for the child’s well-being. The text highlights the tension between maintaining vigilant surveillance, which is culturally associated with ‘good mothering’, and recognising the impact this practice has on the parents’ sleep and well-being. By carefully framing their concerns, the mother opens a dialogue about when it might be appropriate to stop using the monitor, all while ensuring that her actions continue to align with the expectations of responsible mothering.

## Discussion

This study aimed at investigating how mothers account for monitoring their infants’ sleep. We examined how mothers discursively construct their use of digital technologies in online forum and analysed discourses surrounding infants sleep monitoring. Two overarching repertoires were identified which permeated across the data set. The first repertoire was ‘surveillance as a good mothering practice’. This repertoire illustrated how mothers incorporate digital surveillance into their daily routines, using baby monitors as essential tools to enact ‘good mothering’. Mothers’ posts demonstrated how pervasive this surveillance has become, portraying constant monitoring as a normative and responsible behaviour. The second repertoire was ‘risk management via technology’. Mothers discussed ways of limiting risks for their children’s well-being and safety via the use of surveillance technologies. Here, technology was portrayed as a beneficial tool for parents, helping in childcare and enhancing safety. We can locate both repertoires within a wider frame of ‘good mothering’ practices and neoliberal framings of informed choice and risk. The mothers constructed a subject position of ‘good mothers’ using baby monitoring technology; however, the analysis also demonstrated the potential anxiety that the mediatisation of mothering by digital technologies introduces.

This analysis has shown how the discourses of surveillance and risk are pervasive in all mothers’ accounts. It also highlighted how mothers manage their accountability by discussing the ‘good mothering practices’ associated with baby monitor use, which includes extensive digital surveillance of their children. In doing so, it has shown how surveillance is therefore associated as a ‘good mothering practice’, an integral part of managing their accountability regarding risks and safety for their infants. Related to this, baby monitors are framed as ‘risk management’ tools and are associated with ‘good mothering’ practices. However, technology often drives mothering practices, instead of assisting them. Digital surveillance is normalised to the extent that it becomes synonymous with responsible mothering, shaping mothers’ practices and expectations from the earliest stages of a child’s life. These points will now be addressed in turn.

Digital surveillance of their children was presented by mothers as completely normative and average, either with cameras, connected mattresses and baby phones. This was evident in all posts (e.g. post 1 ‘the only thing we have left to purchase is the monitor’ or post 2 ‘the monitor is on and we can see him clearly in it’). This idea that baby monitors will prevent SIDS and other unnamed potential dangers was present in all the posts and resonates with the neoliberal discourse that the use of digital devices monitoring individuals’ health practices will bring a healthier and happier life (Ahmed, 2010; Lupton, 2012). These narratives illustrate how deeply entrenched the association between surveillance and ‘good mothering’ has become, normalising the use of these devices and embedding them in the expectations towards new parents. The visual capabilities of modern baby monitors offer parents a heightened sense of reassurance by enabling continuous observation of their infants. This visual surveillance extends beyond traditional auditory monitoring, allowing parents to visually confirm their baby’s safety and well-being. However, this technological mediation introduces a shift in sensory engagement, as parents rely more on visual data transmitted through devices rather than direct physical interaction. Jewitt et al. (2021) discuss how such digital mediation can reconfigure parent–baby touch, altering traditional notions of connection and parental sensing. The reliance on visual monitoring may lead to a form of ‘knowing touch’ that is less tactile and more observational, potentially impacting the development of intuitive caregiving practices. While these devices provide a sense of control and immediate reassurance, they also contribute to the normalisation of surveillance in parenting, embedding technological observation into the fabric of daily caregiving routines.

Moreover, surveillance served as a means for mothers to manage their accountability, particularly when they were not directly attending to their child. For example, when mothers did something other than caring for the baby, they could be making what is deemed a ‘mothering mistake’ potentially ‘dangerous’. This analysis highlights the significant and often implicit expectations placed on mothers to maintain continuous vigilance over their child from birth. In this context, the baby monitor functions as a tool for managing maternal accountability, enabling mothers to monitor – and thus care for – their child continuously. This is evident in the way they emphasised their ability to ‘see him clearly’ through the monitor (posts 2 and 4), underscoring the device’s role in fulfilling their caregiving responsibilities. This is aligned with neoliberal discourses that encourage individuals to engage in continuous psychological and physical labour, to work towards self-improvement (Riley et al., 2018).

By presenting digital surveillance as consistent with ‘good mothering’ practices, baby monitors were associated with such practices. For mothers, they became an integral part of managing their accountability regarding risks and safety for their infants. For example, in post 2, we read that the mother watches the child from the living room in the evening during a moment of potential ‘relaxation’ by watching him on video. However, paediatrics guidelines stipulate that a child under 6 months should not sleep alone (Moon et al., 2022). Having read this information, the mother wonders whether letting the child sleep alone under the surveillance of the camera is something that other parents do. We can see that using a camera is a way of managing their accountability: the baby sleeps alone but is monitored via the camera. As this shows, the utilisation of digital technology facilitates the separation of infants from their mothers, thereby enabling the infants to be alone in a room. This has the potential to enable mothers to maintain a physical distance from their infants, perhaps because the camera is perceived almost as an extension of the mother’s body. It seems that the guideline is not aligned with contemporary parenting practices. The ‘children should not sleep alone in the first 6 months of life’ instruction lacks clarity and does not facilitate comprehension of its intended implications in a digital world. Hence, mothers may interpret the guideline as ‘do not let the child sleep unsupervised’, which is different. Future guidelines may benefit from taking this digital aspect in consideration and clarify the purpose of this instruction.

The analysis portrayed an evolving frame regarding the ways in which *Mumnseters* discussed the use of digital surveillance over time. While in the first months of a child’s life, baby monitors are presented as essential, reassuring and useful, over time they are seen to have disadvantages. In particular, the injunction to constantly monitor the child was always present, as was the idea that using these tools warns of danger, but the negative facets (baby noises or false alarms) of using these tools gradually became difficult for mothers to bear. At that point, the posts became emotionally charged and relatable, culminating with the idea that the baby monitor is so essential to the child’s safety that removing it for the parent’s comfort puts the baby at risk and could be framed as a mothering mistake in case the baby stops breathing. The analysis showed that digital mothering practices can go beyond the notion of a promise of happiness (Ahmed, 2010) and become a technical imperative that drives mothers rather than assisting them.

## Conclusion

This study explored how discourses of mothering and surveillance are reflected in online discussions about baby monitors. In today’s culture, the widespread focus on risk prevention positions digital surveillance as a key element of ‘good mothering’, framing baby monitors as vital tools for managing risks. Marketed as essential for responsible parenting, these devices often create a physical distance between caregiver and child, transforming traditional, sensory parent-child interactions into technology-mediated ones (Jewitt et al., 2021). This shift aligns with neoliberal ideals that emphasise individual responsibility and self-improvement (Ahmed, 2010; Riley et al., 2018). While baby monitors are intended to help mothers manage their accountability, their pervasive use can inadvertently heighten anxiety and lead to over-surveillance. The analysis further revealed that discontinuing the use of sleep monitors can be challenging, as these devices are often seen as crucial for ensuring infant safety. Mothers also face societal pressure to constantly monitor their children, as gendered expectations equate vigilant oversight with ‘good mothering’, reinforcing unequal gender roles in parental responsibilities.

There are some limitations to this study, particularly the fact that participants are likely familiar with the technology. It would be valuable to investigate the use of baby monitors among both users and non-users, as well as explore other online platforms beyond Mumsnet. Additionally, due to the large volume and variable quality of responses, this study focused solely on the original posts, which is another limitation.

Our findings raise concerns about whether mothers should adapt to these technologies or whether the tools should evolve to better serve parental needs. This calls for a critical reassessment of technology’s role in shaping modern mothering norms, urging policymakers and designers to prioritise tools that supports parents without reinforcing rigid surveillance expectations.
